# Characterization of Phenolic Compounds Extracted from Cold Pressed Cactus (*Opuntia ficus-indica* L.) Seed Oil and the Effect of Roasting on Their Composition

**DOI:** 10.3390/foods9081098

**Published:** 2020-08-11

**Authors:** Malika Chbani, Bertrand Matthäus, Zoubida Charrouf, Hanae El Monfalouti, Badr Kartah, Said Gharby, Ina Willenberg

**Affiliations:** 1 Laboratory of Plant Chemistry and Organic and Bio-Organic Synthesis, Faculty of Sciences, Mohammed V University of Rabat, 10000 Rabat, Morocco; malikachbani96@gmail.com (M.C.); zcharrouf@yahoo.fr (Z.C.); hanae.elmonfalouti@um5.ac.ma (H.E.M.); badreddine.kartah@gmail.com (B.K.); 2 Working Group for Lipid Research, Department of Safety and Quality of Cereals, Max Rubner-Institut (MRI), 32756 Detmold, Germany; bertrand.matthaeus@mri.bund.de; 3 Laboratory Biotechnology, Materials and Environment (LBME), Faculty Polydisciplinary of Taroudant, University Ibn Zohr, 80000 Agadir, Morocco; s.gharby@yahoo.fr

**Keywords:** phenolic compounds, HPLC-ESI-qToF-MS, HPLC-DAD, roasting process, lignin, cactus seed oil

## Abstract

Phenolic compounds extracted from cactus seed oil were identified for the first time by HPLC-ESI-qToF-MS and subsequently quantified by HPLC-DAD. A total of 7 compounds were identified, vanillin, syringaldehyde, and ferulaldehyde were found to be the most abundant ones. The effect of geographical origin and roasting process of cactus seeds was evaluated. Differences between different locations were not found, however the roasting process had a significant effect on the amount of phenolic compounds. The amount of syringaldehyde, *p*-coumaric acid, *p*-coumaric acid ethyl ester, and ferulaldehyde increased during the roasting process. Nevertheless, the concentration of vanillin was not influenced by roasting. It was demonstrated that the increase of those compounds was due to the thermal degradation of lignin from the seeds during the roasting process of seeds.

## 1. Introduction

Cactus (*Opuntia ficus-indica*) is a robust plant belonging to the family *Cactaceae*, originally coming from the arid and semi-arid areas of Mexico. The plant was introduced in North Africa around the 16th century [1]. Today, more than 250 different species are known and distributed in many areas of the world such as America, Africa, but also the Mediterranean Europe, India, and the Middle East [2,3]. In Morocco, especially in areas with limited access to water, the cultivation of cactus plants can play an important role because it is a suitable species for sustainable agriculture and can help to protect the soil against erosion and to fight against the spread of the desert, slows down the decline of fertile soil, and ensures biodiversity. The plant becomes more and more economically important but remains very little exploited. Today, mainly cactus fruits, also called prickly pears, have become very popular for different purposes such as foods, cosmetics, and pharmaceuticals. This generates an income for farmers and local persons in rural areas of Morocco and therefore plays an important socio-economic role and contributes to a sustainable development in rural areas.

Cactus plants occupy an area of over one hundred thousand hectares in Morocco, with a national fruit production estimated as about 1.1 million tons, of which 24% come from Kelaa Sraghnas, 24% from Tiznit, 19% from Guelmim, and 5% from Chtouka Ait Baha. The annual production of cactus fruits can reach 50 tons/hectare [4,5]. *Opuntia ficus-indica* is the most cultivated species of the genera Opuntia and it can be found almost everywhere in Morocco. The cultivation of *O. ficus-indica* is low in investment and it can generate an important income by using the different parts of the plant for the preparation of food for human nutrition.

Cactus fruit (prickly pear) is a many-seeded berry with a thick peel which includes the flavored seed-rich pulp. The fruit contains around 300 seeds [6] and the weight of the seeds can be between 30 and 40% of the dried fruit [7]. Until recently, these seeds were discarded as waste, although this by-product is an important source for an valuable oil and also the press cake is usable in human nutrition or animal feed [8,9]. The further use of the seeds allows, on one side, to reduce the amount of waste and improves the waste management, and on the other side, a sustainable comprehensive use of fruits becomes possible. At present, cactus seed oil has been presented as a new ingredient in the field of the high-value cosmetic ingredients primarily due to its specific chemical composition ideal for cosmetic applications [10,11,12].

The cactus seeds are small with a weight between 15 and 20 mg [13] and a low oil content between 5 and 15%, compared to conventionally used oil seeds such as rapeseed (45%) or sunflower seed (20%), but a valuable fatty acid composition with linoleic acid as the main component [14,15]. The oil obtained from the seeds by screw-pressing and filtration for the clean-up is mainly used in cosmetics due to the high price and the time-consuming and laborious process of production resulting from the small kernels. On the other side, the oil is also suitable for human consumption, with a fatty acid composition similar to sunflower oil and γ-tocopherol as the main antioxidant compound [11,16]. The most important group among the minor components of cactus are phenolic compounds that may act as antioxidants against oxidative damage but are also described as compounds with a positive effect on health [17,18]. Phenolic compounds are strongly correlated to the antioxidant activity and several reports showed a linear correlation between antioxidant activity and the content of phenolic compounds. For the phenolic compounds, several positive health effects have been described including anticancer [19,20], antimutagenic [21], antimicrobial [22], antidiabetic [23], and anti-inflammatory [24] properties. Therefore, the knowledge of the composition of phenolic compounds in cactus seed oil is of great interest.

Over the last years, there has been an abundance of scientific papers on cactus pear and especially the seeds were identified as raw material with high amounts of phenolic compounds [7,25,26], underlining the interest in the numerous properties (both its bioactivity and coloring potential) of this plant species, well adapted to extreme growing conditions in arid and semi-arid zones. However, no information is available regarding the content and composition of phenolic compounds extracted from the oil during cold-pressing of cactus seeds.

Therefore, the aim of the present work was to characterize, for the first time, the phenolic compounds extracted from cactus seed oil obtained from six different geographical origins in Morocco. In the future, this information could be used to verify the authenticity of cactus seed oil similar to other vegetable oils. Results already published for the composition of phenolic compounds in fruits or seeds are of limited use only, since during oil extraction, most of the phenolic compounds remain in the press cake, resulting in a shift in the composition of the phenolic compounds. In addition, the effect of roasting seeds at 110 ± 5 °C for 10 to 40 min on the content of phenolic compounds was investigated. Roasting of seeds is a common procedure for many different kinds of oilseeds to give the oil a pleasant taste and smell. Examples are roasting of rapeseed, sesame seeds, argan almonds, etc. Depending on the roasting conditions, Maillard reaction products are formed that are perceived positively by the consumer. Therefore, the aim of the present work was to get an idea about the fate of phenolic compounds as a result of roasting.

## 2. Materials and Methods

### 2.1. Samples

**Figure 1:**
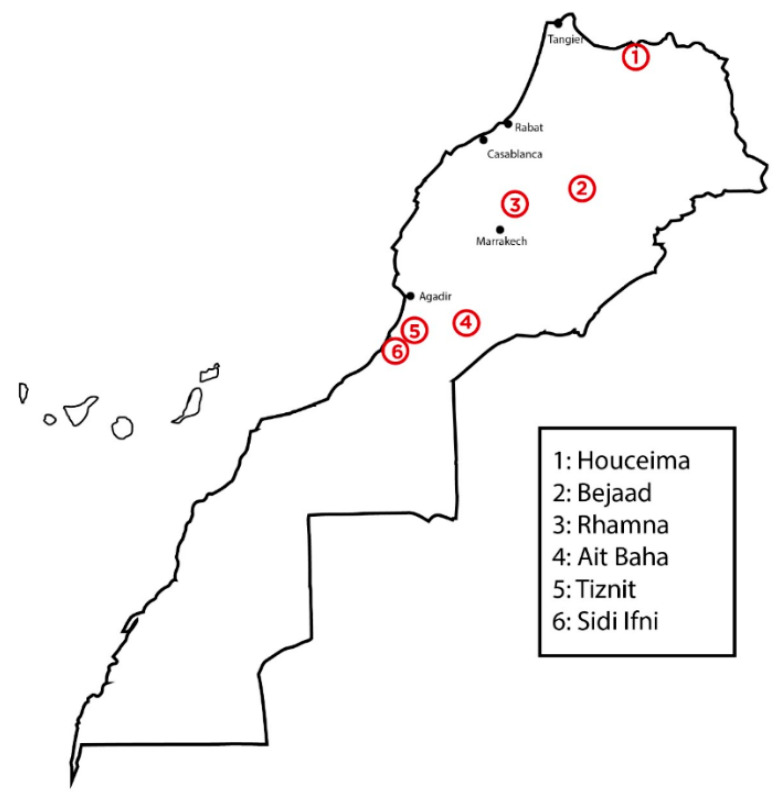
Location of the evaluated cactus seed sites of production in Morocco.

Cactus seeds used for this investigation were harvested between June and August 2017 at different locations in Morocco: Bejaad (32°46′15″ N, 6°23′28″ W; elevation: 680 m), Ait Baha (30°4′7.9″ N, 9°9′10″ W; elevation: 604 m), Rhamna (32°28′12″ N, 7°57′29″ W; elevation: 491 m), Tiznit (29°42′1″ N, 9°43′43″ W; elevation: 252 m), Houciema (35°14′41″ N, 3°55′60″ W, elevation: 133 m), and Sidi Ifni (29°22′45″ N, 10°10′17.6″ W; elevation: 0 m) (Figure 1).

For the study of the effect of roasting on the content and composition of phenolic compounds, only seeds from Sidi Ifni were used. Roasting was carried out at 110 ± 5 °C for 10, 20, 30, or 40 min using a roaster with continuous mixing of the material. The temperature was monitored using a Testo 945 thermometer sensor (Testo, Casablanca, Morocco). The extraction of the oil was carried out using a CA59 G screw press (IBG Monforts GmbH & Co., Mönchengladbach, Germany). After extraction, the oil was left in the fridge over night to allow plant particles to settle. Afterwards, the oil was centrifuged for 15 min at 3000 rpm and the oil was separated from the sediment. The purified oil was stored in the fridge (4 °C) until analysis.

### 2.2. Reagents

As analytical standards for the identification of the distinct phenolic compounds in cold pressed cactus seed oil samples, the following compounds were purchased from Sigma-Aldrich (St. Louis, MO, USA): 4-hydroxy benzaldehyde, vanillin, syringaldehyde, *p*-coumaric acid, ferulic acid, ferulaldehyde, and *p*-coumaric acid ethyl ester. HPLC grade methanol (Baker, Avantor Performance Materials Poland S.A.) was used for the extraction of the phenolic compounds from cactus seed oil samples and as mobile phase for the HPLC. Phosphoric acid, acetonitrile, and formic acid for the mobile phase were supplied from Sigma-Aldrich (St. Louis, MO, USA). For the isolation of lignin from cactus seeds, toluene was purchased from Th. Geyer (Renningen, Germany), technical ethanol was distilled, and sulfuric acid was obtained from Merck (Darmstadt, Germany).

### 2.3. Extraction of Phenolic Compounds

The extraction of phenolic compounds from cactus seed oil was carried out by liquid–liquid extraction. In brief, 2 g of sample material were exactly weighed into a 10 mL glass tube, after adding 5 mL of MeOH/H_2_O (80/20 (v/v)), the sample was shaken for 1 min (Vortex, 1500 Mot/min), then centrifuged for 15 min at 3000 rpm. After centrifugation, the methanol phase was removed by a pasteur pipette, and the solvent transferred to a new flask. The extraction process was repeated a second time with the remaining part of oil for a comprehensive extraction of the oil. The second extract was combined with the first one. Finally, the solvent of the combined extract was removed under nitrogen at 40 °C, and then the residue was dissolved in 500 μL MeOH/H_2_O (50/50 (v/v)). The dissolved extract was shaken for 1 min (Vortex (1500 Mot/min)), filtrated through a syringe filter (PTFE 0.2 μm, WICOM Germany GmbH, Heppenheim, Germany), and then transferred in a vial to be injected in the HPLC-DAD or HPLC-ESI-qToF-MS system.

### 2.4. HPLC-DAD Analysis

The analysis of phenolic compounds extracted from cold pressed cactus seed oil was performed using a HPLC-DAD system (VWR, Hitachi, Darmstadt, Germany), equipped with a reversed phase C18 column (250 × 4 mm, i.d. 5 μm, LichroCART, Lichrospher, Merck, Darmstadt, Germany). During the analysis, the column temperature was set to 40 °C. The mobile phase degassed by ultrasonic treatment was A: water/phosphoric acid (99.5/0.5, (v/v)) and B: methanol/acetonitrile (1/1, (v/v)). The composition of the gradient was: 5% of B at the beginning (0 min) and then changed to obtain 30%, 38%, 45%, 52.5%, 100% and 5% B at 15, 30, 40, 45, 50, and 60.1 min, respectively, in a total run time of 65 min.

The flow rate was 1 mL/min and the injection volume was 20 μL. The detection was conducted on a diode array detector L-2455 (Merck-Hitachi, Darmstadt, Germany) at the wavelengths 280, 310, and 335 nm. EZ Chrom Elite (VWR International GmbH, Darmstadt, Germany) was used as software for the acquisition and evaluation of the data.

### 2.5. HPLC-ESI-qToF-MS Analysis

The analysis of the polar extract by a high-performance liquid chromatography-electrospray ionization-quadruple time of flight mass spectrometer (HPLC-ESI-qToF-MS) was made with a HPLC UltiMate 3000 (Thermo Fisher Scientific, Sunnyvale, CA, USA) equipped with an autosampler, in which the samples were stored at 5 °C. Separation was carried out at 40 °C with a 150 mm × 2.1 mm, 1.7 μm Kinetex column EVO C18 (Phenomenex, Torrance, CA, USA). The mobile phase was A = water with 0.01% HCOOH and B = MeOH with 0.01% HCOOH. The percentage of organic modifier (B) was changed as follows: 0 min, 10%; 5 min, 30%; 14 min, 60%; 14.50 min, 70%; 14.51 min, 100%; 16.00 min, 100%; 16.01 min, 10% in a total run time of 18 min. The injected volume was 10 μL. The detection was conducted on a Maxis Impact HD (Bruker Daltonik, Bremen, Germany) in MS/MS (broadband collision induced dissociation (bbCID)) mode following negative electrospray ionization and additionally the diode array detector was used at the wavelength 235, 280, and 335 nm. Nitrogen was used as a desolvation gas and nebulizing gas. MS data were acquired over an m/z range of 50–1000. The values of the other parameters were set in the following way: capillary voltage 3000 V; drying gas temperature 200 °C; dry gas flow 8 L/min; nebulizing gas pressure 2 bar and a plate offset −500 V. A calibration solution of sodium formate (10 mM) was infused to the MS for the first 0.5 min of every run (flow: 0.18 μL/min).

Compass of Series 1.7 package was used as software for the acquisition of the data. After every ten injections, a blank MeOH/H_2_O (10/90 (v/v)) was used to check the presence of contaminations in the system.

### 2.6. Isolation of Klason Lignin from Cactus Seed

For the extraction of Klason lignin from cactus seeds, the following method was used with some modifications [27,28]. In brief, 20 g of cactus seeds were used for the successive extraction with three different solvents in a Soxhlet apparatus with 200 mL of solvent each: 1st extraction with an ethanol-toluene mixture (1:2, v/v) (12 h); 2nd extraction with ethanol (12 h); 3rd extraction with water (12 h). The samples were then dried for 2 days in a hood at room temperature and then in an oven at 40 °C for 24 h. The final residue obtained is called “parietal residue”.

Then, 3 g of the parietal residue was placed in the presence of 40 mL of 72% H_2_SO_4_ (w/w), in a 100 mL beaker for hydrolysis. After mixing the solvent with the solid material using a glass rod, the hydrolysis container was left at room temperature for 2 h, with manual stirring with the glass rod at regular intervals. For post-hydrolysis, the suspension was diluted with distilled water, to bring the solution to a concentration of 3% in H_2_SO_4_. The mixture was then boiled under reflux for 3 h. After cooling, the solution was filtered on a crucible with a previously tared filter and washed until the filtrate was pH neutral. The insoluble residue was washed with distilled water in a Twisselmann apparatus for 2 h and then dried in an oven at 40 °C overnight. The final residue is called Klason lignin.

One part of the Klason lignin was heated for 40 min at 105 °C and the other part not, then both residues were extracted according to the same extraction method used for extraction of phenolic compounds from cactus seed oil. Finally, the extract was injected in the HPLC-DAD (0.5 g of extract in 250 µL MeOH/H2O (50/50, (v/v), 20 μL injection volume).

### 2.7. Data Analysis

The analysis of the HPLC-ESI-qToF data was performed by using Data Analysis (Bruker). The mass to charge ratio (m/z) of the molecular ion peak [M-H]^−^ of the questionable compounds was identified with the help of the MS spectra. The software tool “3D smart formula” was used to calculate potential sum formulas. A database of phenolic compounds called Phenols Explorer (INRA, Paris, France) was used to identify phenolic compounds that have the same formula.

EZ Chrom Elite was used for evaluation and integration of the peaks obtained by HPLC-DAD analysis. Identification of the components was done by comparison of retention time and UV spectra with that of the analytical standards which were chosen based on the assumptions received from the HPLC-ESI-ToF-MS experiments.

For the quantitative analysis, the samples were analyzed in triplicate, exception for lignin in duplicate, the results were expressed as mean ± standard deviation. Quantification was carried out by external calibration. 4-hydroxy benzaldehyde, vanillin, syringaldehyde, *p*-coumaric acid, ferulic acid, and ferulaldehyde in a concentration of 0.3 mg/L, 1.0 mg/L, 2.5 mg/L, 5.0 mg/L, 10.0 mg/L, 15.0 mg/L, 25.0 mg/L, and 50.0 mg/L were used for the calibration. Quantification of *p*-coumaric acid ethyl ester was done by use of a calibration curve prepared of *p*-coumaric acid.

### 2.8. Statistical Analysis

The statistical analysis of the results obtained from cactus seed oil from roasted seeds was carried out by one-way ANOVA followed by Tukey–Kramer post-hoc test, *p* < 0.05 were considered as significant (JMP 14.3.0, SAS Institute Inc., Cary, North Carolina, USA). For the comparison of Klason lignin without and after heat treatment, unpaired Student’s-*t* test was applied.

## 3. Results and Discussion

### 3.1. Phenolic Compounds

Natural phenolic compounds have received increasing interest in the last years. They are constituted in one of the biggest and widely distributed groups of secondary metabolites present in plant material, stressing among them the flavonoids, tannins, chalcones, coumarins, and phenolic acids.

Another interesting aspect is the use of minor components such as phenolic compounds for the verification of authentication of vegetable oils [29]. Especially, expensive oils like cactus seed oil are suspicious to be subjected to different kinds of food fraud. Different types of vegetable oils have very characteristic fingerprints of phenolic compound patterns that can be used to differentiate different oils, mixtures of oils, but also identify the origin of oils [30,31]. Therefore, it is necessary to know more about the composition of phenolic compounds in cactus seed oil. Several papers on the composition of fruits and seeds are known, but these results are not transferable to the oil, because during oil extraction by a screw press, the major part of the phenolic compounds remains in the press cake and thus the composition in the oil is significantly different.

Recently, research has been directed towards natural antioxidants in the seeds of *O. ficus-indica* which were shown to be rich in polyphenols, flavonoids, and tannins with a higher concentrations of those molecules than in the fruit pulp [7].

**Figure 2:**
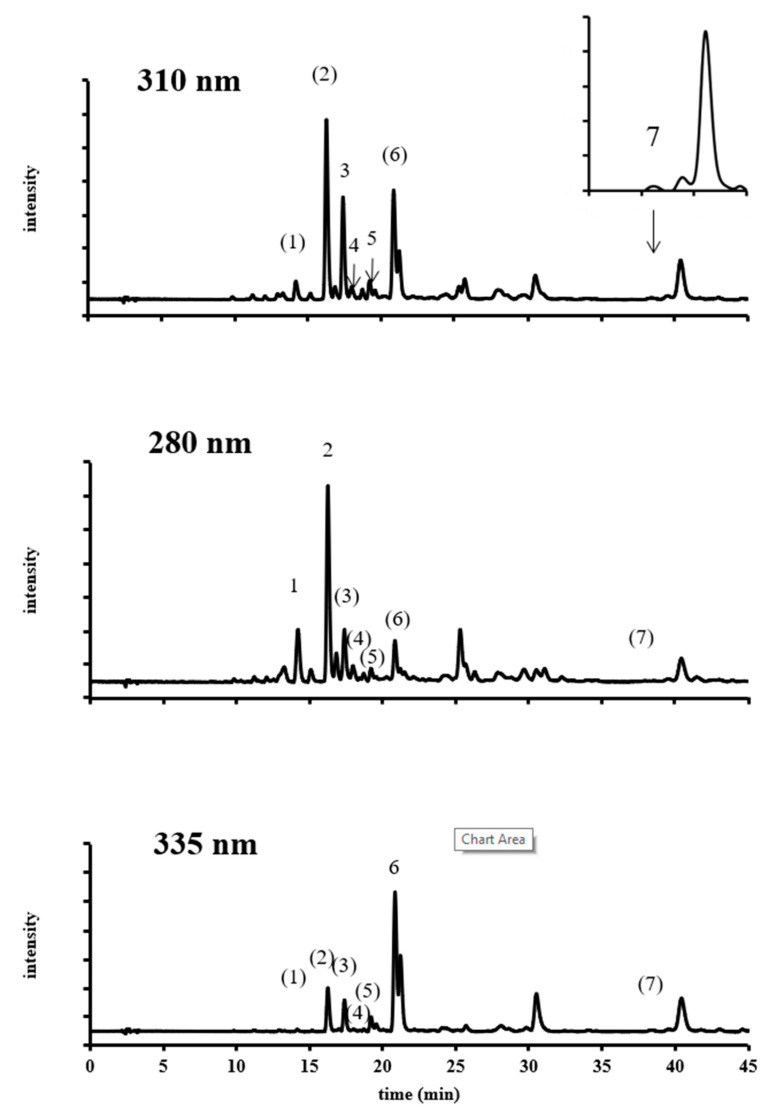
Chromatogram of a cactus seed oil sample. (1) 4-hydroxy benzaldehyde; (2) vanillin; (3) syringaldehyde; (4) *p*-coumaric acid; (5) ferulic acid; (6) ferulaldehyde; (7) *p*-coumaric acid ethyl ester. Compounds with numbers within brackets were quantified with another wavelength.

**Figure 3:**
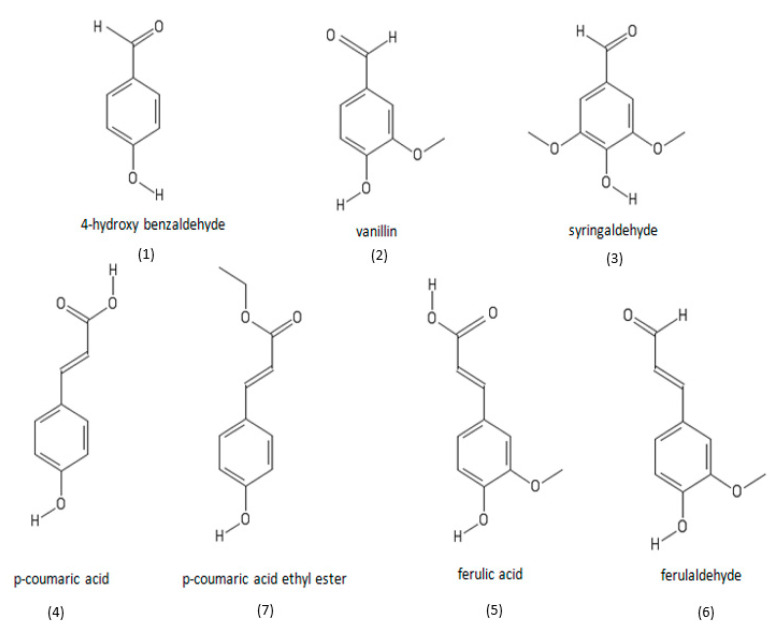
Structural formulas of phenolic compounds identified in cactus seed oil.

In order to identify phenolic compounds present in the oil, the qualitative analysis of *O. ficus-indica* seed oil was performed by HPLC-DAD and HPLC-ESI-qToF-MS. A typical HPLC-DAD chromatogram of cactus seed oil is shown in Figure 2. As it is often not possible to identify compounds only by the help of the UV-spectra, the information derived by the MS and MS/MS spectra was additionally used to develop suggestions for the structure of the observed signals. Finally, the suggestions were verified by the analysis of the distinct reference standards. As an example, the MS and MS/ MS spectra of ferulaldehyde showed that the most abundant signal at the retention time of 7.3 min was m/z = 353.1. According to the 3D formula tool, possible formulas were found to be C_10_H_10_O_3_ or C_20_H_18_O_6_. Based on this sum formula, potential phenolic compounds were identified with the Phenol explorer database as ferulaldehyde, mellein, sesamine, or episesamine. Based on the MS/MS and UV-spectra of the potential compounds, the most probable assumption was found to be ferulaldehyde. For that, the analytical reference standard of ferulaldehyde was injected in HPLC-DAD and the retention time and UV spectra were compared with those of the peak, which finally fitted to each other. Therefore, the presence of ferulaldehyde in cactus seed oil could finally be concluded. This approach allowed to identify seven phenolic compounds (Figure 3) belonging to three families: hydroxybenzaldehyde derivates (4-hydroxy benzaldehyde, vanillin, syringaldehyde), hydroxyl cinnamic acid derivates (*p*-coumaric acid, *p*-coumaric acid ethyl ester, ferulic acid), and hydroxyl cinamaldehyde derivates (ferulaldehyde).

To the best of our knowledge, no information is available about the composition and the concentration of phenolic compounds in cactus seed oil. Therefore, in the following, other publications dealing with the composition of phenolic compounds from cactus seeds and fruits were used. Zenteno-Ramirez et al. [32] found gallic acid, syringic acid, and ellagic acid as main phenolic acids in lyophilized juices of 10 prickly pear variants, with gallic acid as the predominant component. None of these compounds were found in the present investigation in the oil. They also found in the juice, higher amounts of flavan-3-ol derivatives, such as catechin, epicatechin, procyanidin B1, and Procyanidin B2. Kivrak et al. [33] found 19 phenolic compounds in fruits of *O. ficus-barbarica*, A. Berger and *O. robusta*, J.C. Wendl with highest amounts for ferulic acid (26.9 and 31.6 mg/kg) and *p*-coumaric acid (8.6 mg/kg) in *O. ficus-barbarica*. In contrast to these results, Kim et al. [34] isolated nine flavonoids and four simple phenolic glycosides from *O. ficus-indica fruits*, such as 1-O-feruloyl-β-D-glucopyranoside, isorhamnetin 3-O-β-D-glucopyranoside, kaempferol 3-methyl ether, kaempferol, or quercetin. All these compounds are only limited soluble in the oil. The absence of these compounds in oil can be explained by the fact that some of them are glycosides that more likely remain in the press cake instead of being extracted in the oil due to the higher polarity of the compounds. Santos et al. [35] extracted the cladode peels of *O. ficus-indica* with a mixture of alcohol and water and finally identified fifteen compounds in the resulting extract such as methyl, glycosylated and aglycone quercetin derivatives, and aglycone and glycosylated kaempferol derivatives. It can be assumed that most of these compounds are soluble in the oil due to their structure. Only small amounts of vanillin and *p*-coumaric acid were found by Allai et al. [36] in cladodes of *O. ficus-indica* collected from the Moroccan Settat region after extraction with ethanol or acetone, respectively. The main phenolic compounds identified in the extracts were quinic acid and malic acid, as well as some flavonoids such as rutin, quercitin, or kampferol.

There are limited publications about the identification of phenolic compounds from *O. ficus-indica* seeds. Chougui et al. [7] identified feruloyl derivatives, mainly glycosides and sinapoyl-diglucoside. These compounds are more water soluble due to at least one sugar moiety and therefore should mainly remain in the press cake during oil processing. However, hydrolysis as a result of the pressing process could led to the formation of the aglycons. Thus, the detection of ferulic acid and ferulaldehyde in the seed oil is in line with these previously described observations of feruloyl derivates in the seeds. Isorhamnetin was identified by Chahdoura et al. [37] in *Opuntia microdasys* seeds from Tunisia. The compound belongs to the group of flavonoids. Tounsi et al. [25] detected in methanolic macerate as major compounds of cactus seeds of ripe fruits catechin (61.0%) and rutin (10.1%). Both compounds are soluble in methanolic solution but not in the oil. Therefore, these compounds were probably not found in the present investigation.

### 3.2. Investigation of Phenolic Compounds of Cactus Seed Oil by Region

The amount of seven phenolic compounds identified in cactus seed oils from seeds of different regions of Morocco was determined and the results are shown in Table 1. The results obtained show that the major phenolic compounds in all oils from different regions were vanillin, syringaldehyde, and ferulaldehyde. The highest amount of those compounds was found in Bejaad (32.4, 12.3, and 5.7 mg/kg, respectively), followed by Sidi Ifni (18.5, 8.3, and 4.6 mg/kg, respectively). In addition, the oils from both locations (Bajaad and Sidi Ifni) are characterized by a significant higher amount of vanillin and 4-OH benzaldehyde in comparison to the oils from the other locations (Table 1). On the other hand, oil from Bejaad also contained a significant higher amount of syringaldehyde. The amount of the other compounds in the oils was comparable. The differences in the composition of cactus seed oils from different locations may be mainly attributed to genetic diversity, weather conditions, the harvest season, storage conditions, or processing, because these factors strongly affect the profiles of secondary metabolites [38].

Despite significant differences between oils of some locations, a final statement whether those compounds are characteristic for different regions is difficult in the current stage of the investigation as only one sample per region was analyzed. For this, more and different samples from those regions are needed to be investigated. However, all analyzed oils from the different locations show a very similar pattern of phenolic compounds that could serve as a fingerprint to ensure the authenticity of cactus seed oil.

Interesting is the high content of vanillin in cactus seed oil obtained from seeds cultivated in Bejaad and Sidi-Ifni. Vanillin is one of the most important edible flavors, but some studies show that vanillin may also act as an antioxidant in complex foods containing polyunsaturated fatty acids [39]. Moreover, syringaldehyde is one of the less explored phenolic compounds, and so far, there is little information on its antimicrobial activity, as well as its other properties. Ferulic acid and ferulaldehyde are potential end-products of dietary polyphenol degradation [40,41]. Furthermore, ferulic acid was reported to stay in the blood longer than other antioxidants such as vitamin C, and have higher bioavailability [40]. Due to the structural similarity between ferulic acid and ferulaldehyde, and the presence of the reactive aldehyde group (which can be easily oxidized to a carboxylic group), ferulaldehyde is thought to have very similar or maybe better biological activity as ferulic acid [42].

### 3.3. Investigation of Phenolic Compounds of Cactus Seed Oil Obtained from Seeds Roasted for Different Times

**Figure 4:**
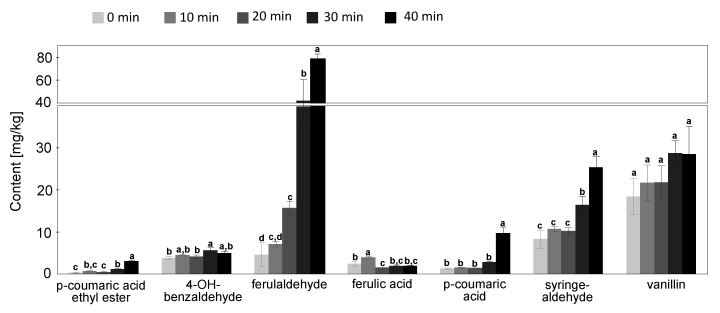
Changes of phenolic compounds composition during roasting process shown as concentration in mg/kg (mean ± SD, analytical replicate = 3). Values with different letters for each compound are significantly different in comparison to the other time points by applying Tukey–Kramer HSD test (*p* < 0.05).

The samples from the roasting process were analyzed to determine the effect of the roasting time on the amount of phenolic compounds identified. The results obtained are shown in Figure 4.

The statistical test shows that the roasting time has no influence on the amount of vanillin in the oil but with increasing roasting time, the amount of syringaldehyde, *p*-coumaric acid, ferulaldehyde, and *p*-coumaric acid ethyl ester in cactus seed oil increased. The increase of phenolic compounds in cactus seed oil resulting from roasting from 0 to 40 min at 110 °C led to amounts between 8.3 ± 2.2 and 25.4 ± 2.5 mg/kg (syringaldehyde), 1.3 ± 0.2 and 9.7 ± 1.5 mg/kg (*p*-coumaric acid), and 4.6 ± 2.8 and 76.3 ± 6.4 mg/kg (ferulaldehyde) and 0.2 ± 0.2 and 3.1 ± 0.2 mg/kg (*p*-coumaric acid ethyl ester). The most significant increase for syringadehyde and ferulaldehyde was found for a roasting time of 30 and 40 min, respectively, while for *p*-coumaric acid and *p*-coumaric acid ethyl ester, a significant increase was found at a roasting time of 40 min.

The increase of the concentration of those phenolic compounds during roasting might be explained by heat-catalyzed hydrolytic reaction of its native esterified or bound forms liberating into free forms. Naturally, polyphenols occur as in both free and bound forms [43]. Some processing methods such as roasting have been shown to increase the amount of phenolic compounds probably due to releasing free phenolic compounds from the bound forms and coupled with formation of Maillard reaction products due to roasting effects [43]. Further, the disruption of the cell wall through heating or by the breakdown of insoluble phenolic compounds as function of thermal treatments could lead to a better extractability of phenolic compounds [43]. This explanation is supported by the composition of the phenolic compounds of the oil. Lignin is mainly composed of *p*-coumaril, coniferyl (= feruloyl), and sinapyl units [44]. It might be that the increase of phenolic compounds during roasting is attributed to thermal degradation of lignin.

Some studies on the effect of roasting on the content of phenolic compounds and antioxidant activities of whole cashew nuts, kernels, and testa show that the content of phenolic compounds increased with increasing temperature [45]. The same results were found for roasting of sesame seeds at 200 °C for 60 min and also for pumpkin seed oil, that show a significant increase of total phenolic compounds due to the roasting process [45,46]. For proso millet (*Panicum miliaceum*), Han et al. [47] also showed that roasting increased the content of phenolic compounds. 

It can be concluded that the roasting process can be used to increase the amount of some phenolic compounds in cactus seed oil. That might lead to an increase of the antioxidant activity of the formed compounds. Finally, this could result in an efficient ways to prevent lipid oxidation. Further investigations, especially to the effect of roasting on the antioxidant activity of cactus seed oil, are necessary.

### 3.4. Release of Phenolic Compounds from Cactus Seeds during Roasting

Lignin constitutes, after cellulose, the second biopolymer most abundant terrestrial, approximately 30% of the organic carbon in the biosphere [48]. Lignin is a phenolic biopolymer derived from hydroxy cinnamyl alcohols that differ in their degree of methoxylation: *p*-coumaryl, coniferyl, and sinapyl alcohols. It is formed under simple chemical control by bimolecular radical coupling reactions and its structure is highly dependent on the nature of monolignols and the cellular characteristics of the lignified tissue [49].

**Figure 5:**
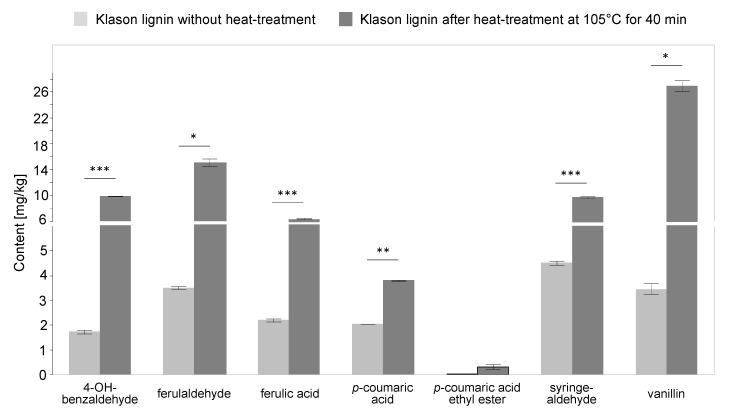
Changes of phenolic compounds composition during thermal treatment (40 min at 105 °C) of isolated Klason lignin from cactus seeds, shown as concentration in mg/kg (mean ± SD, *n* = 2). Statistically significant differences between Klason lignin without and after heat-treatment were determined by unpaired Student’s-t test (* *p* < 0.05, ** *p* < 0.01, *** *p* < 0.001).

The aim of the isolation of Klason lignin from cactus seeds was to study the effect of thermal treatment on the degradation of lignin and the formation of free phenolic compounds. This should help to explain the increase of some phenolic compounds during roasting. The results obtained show, in Figure 5, an increase of the phenolic compounds due to the thermal treatment.

A significant increase of the phenolic compounds extracted from thermal treated Klason lignin from cactus seeds in comparison to untreated Klason lignin was revealed for all phenols except for *p*-coumaric acid ethyl ester, of which increase was not found to be significant. From this, it can be concluded that the increase of those phenolic compounds in the oil from roasted seeds is due to the thermal degradation of lignin. A remarkable increase was observed especially for vanillin, ferulaldehyde, and syringaldehyde. This result is in accordance with the literature, which shows that a dehydration of the lignin structure results in pyrolysis products with unsaturated side chains, such as vanillin and vanillic acid, coniferyl-(cis-trans) and dihydroconiferyl alcohol, coniferaldehyde and sinapaldehyde, *p*-hydroxycinnamic alcohols [44]. Another study on the thermal degradation of softwood and hardwood lignin revealed that guaiacol-type and syringol-type compounds are the dominant products for both two lignins at lower temperature, while the amount of phenol-type compounds, catechol-type compounds, and aromatic hydrocarbons was notably increased at an elevated temperature [50].

## 4. Conclusions

During the last decade, growing interest in *Opuntia ficus-indica* has resulted in a large number of scientific papers. For the best of our knowledge, all of those studies dealt with fruits or seeds of the prickly pear. However, there are only few studies taking the cactus seed oil into account.

In this study, *O. ficus-indica* seed oil from six regions of Morocco and from different roasting times was used for the first time to investigate the phenolic compounds and the effect of roasting on its amount. The composition of phenolic compounds of cactus seed oil is mainly characterized by vanillin, syringaldehyde, and ferulaldehyde, with some influence of the location on the content of phenolic compounds. The pattern of the phenolic compounds is comparable between the oils from seeds obtained from different locations. Therefore, it would be very useful to further investigate the application of a fingerprint of phenolic compounds from cactus seed oil to detect adulteration of this very expensive vegetable oil. For this, a larger number of samples of cactus seed oil has to be investigated regarding the composition and the pattern of phenolic compounds.

Mild roasting of the seeds increases the total content of the phenolic compounds extracted with the oil from the seeds. After the roasting process, higher amounts of *p*-coumaric acid ethyl ester, vanillin, feruladehyde, *p*-coumaric acid, and syringaldehyde were found in the oil. The results of the present paper show that this increase in phenolic compounds may be attributed to the thermal degradation of lignin located especially in the shell of the seeds.

## Figures and Tables

**Table 1:** Phenolic compounds composition of cactus seed oil from different region of Morocco expressed in mg/kg and shown as mean ± SD (*n* = 3). Statistical difference was determined by one-way ANOVA followed by Tukey post-hoc test. Different letters in the same line comparing the regions represent statistically significant results at 95% confidence interval.

Compound	Houceima	Bejaad	Rhamna	Ait Baha	Tiznit	Sidi Ifni
4-OH benzaldehyde	1.1 ± 0.01 ^b,c^	3.7 ± 0.05 ^a^	1.4 ± 0.01 ^b^	0.9 ± 0.02 ^c^	1.0 ± 0.01 ^b,c^	3.8 ± 0.37 ^a^
vanillin	6.7 ± 0.03 ^c^	32.4 ± 0.68 ^a^	6.8 ± 0.02 ^c^	3.9 ± 0.04 ^c^	4.4 ± 0.05 ^c^	18.5 ± 4.33 ^b^
syringaldehyde	5.6 ± 0.06 ^c^	12.3 ± 0.14 ^a^	4.1 ± 0.05 ^c,d^	2.4 ± 0.19 ^d^	2.3 ± 0.02 ^d^	8.3 ± 2.16 ^b^
*p*-coumaric acid	0.7 ± 0.01 ^c,d^	2.2 ± 0.04 ^a^	0.8 ± 0.02 ^c^	0.6 ± 0.11 ^c,d^	0.5 ± 0.01 ^d^	1.3 ± 0.16 ^b^
ferulic acid	0.7 ± 0.09 ^b^	1.0 ± 0.05 ^b^	1.1 ± 0.03 ^b^	0.7 ± 0.10 ^b^	0.9 ± 0.02 ^b^	2.4 ± 0.53 ^a^
ferulaldehyde	4.9 ± 0.04 ^a^	5.7 ± 0.04 ^a^	3.6 ± 0.01 ^a^	2.8 ± 0.02 ^a^	2.6 ± 0.03 ^a^	4.6 ± 2.84 ^a^
*p*-coumaric acid ethyl ester	0.2 ± 0.12 ^a^	0.1 ± 0.02 ^a^	0.5 ± 0.15 ^a^	0.3 ± 0.23 ^a^	0.2 ± 0.12 ^a^	0.2 ± 0.18 ^a^

## References

[1] Griffith M.P. (2004). The origins of an important cactus crop, *Opuntia ficus-indica* (*Cactaceae*): New molecular evidence. Am. J. Bot..

[2] Curtis J.R. (1977). Prickly pear farming in the Santa Clara Valley, California. Econ. Bot..

[3] Brutsch M.O., Zimmermann H.G. (1993). The prickly pear (*Opuntia ficus-indica* [*Cactaceae*]) in South Africa: Utilization of the naturalized weed, and of the cultivated plants. Econ. Bot..

[4] Domínguez López A. (1995). Review: Use of the fruits and stems of the prickly pear cactus (*Opuntia* spp.) into human food. Food Sci. Technol. Int..

[5] El Kossori R.L., Villaume C., El Boustani E., Sauvaire Y., Mejean L. (1998). Composition of pulp, skin and seeds of prickly pears fruit (*Opuntia ficus indica* sp.). Plant Foods Hum. Nutr..

[6] Barbera G., Inglese P., La Mantia T. (1994). Seed content and fruit characteristics in cactus pear (*Opuntia ficus-indica Miller*). Sci. Hortic..

[7] Chougui N., Tamendjari A., Hamidj W., Hallal S., Barras A., Richard T., Larbat R. (2013). Oil composition and characterisation of phenolic compounds of Opuntia ficus-indica seeds. Food Chem.

[8] Barba F.J., Putnik P., Bursać Kovačević D., Poojary M.M., Roohinejad S., Lorenzo J.M., Koubaa M. (2017). Impact of conventional and non-conventional processing on prickly pear (*Opuntia* spp.) and their derived products: From preservation of beverages to valorization of by-products. Trends Food Sci. Technol..

[9] Habibi Y., Heux L., Mahrouz M., Vignon M.R. (2008). Morphological and structural study of seed pericarp of Opuntia ficus-indica prickly pear fruits. Carbohydr. Polym..

[10] Guillaume D., Gharby S., Harhar H., Baba M. (2015). Opuntia ficus-indica and Balanites aegyptiaca oils: Two seed oils to watch. Hausehold Pers. Care Today.

[11] Taoufik F., Zine S., El Hadek M., Hassani L.M.I., Gharby S., Harhar H., Matthaus B. (2015). Oil content and main constituents of cactus seed oils Opuntia ficus-indica of different origin in Morocco. Mediterr. J. Nutr. Metab..

[12] Ciriminna R., Bongiorno D., Scurria A., Danzi C., Timpanaro G., Delisi R., Avellone G., Pagliaro M. (2017). Sicilian Opuntia ficus-indica seed oil: Fatty acid composition and bio-economical aspects. Eur. J. Lipid Sci. Technol..

[13] Khan D. (2006). Some seed and seedling characteristics (tricotyledony) of *Opuntia ficus indica* (L.) Mill. (*Cactaceae*). Int. J. Biol. Biotechnol..

[14] Ghazi Z., Ramdani M., Fauconnier M.L., Mahi B.E., Cheikh R. (2013). Fatty acid, sterol and vitamin E composition of seed oil of Opuntia Ficus-Indica and Opuntia Dillenii from Morocco. J. Mater. Environ. Sci..

[15] Ennouri M., Evelyne B., Laurence M., Hamadi A. (2005). Fatty acid composition and rheological behaviour of prickly pear seed oils. Food Chem..

[16] Ramadan M., Mörsel J.-T. (2003). Oil cactus pear (*Opuntia ficus-indica* L.). Food Chem..

[17] Bhuyan D.J., Basu A. (2017). Phenolic Compounds: Potential Health Benefits and Toxicity Deep Jyoti Bhuyanand Amrita Basu.

[18] Vermerris W., Nicholson R. (2008). Phenolic Compounds and their Effects on Human Health.

[19] Carocho M., Ferreira I.C.F.R. (2013). The role of phenolic compounds in the fight against cancer—A review. Anti Cancer Agents Med. Chem..

[20] Basli A., Belkacem N., Amrani I. (2017). Health Benefits of Phenolic Compounds Against Cancers. 10.

[21] Makhafola T.J., Elgorashi E.E., McGaw L.J., Verschaeve L., Eloff J.N. (2016). The correlation between antimutagenic activity and total phenolic content of extracts of 31 plant species with high antioxidant activity. BMC Complement. Altern. Med..

[22] Bouarab-Chibane L., Forquet V., Lantéri P., Clément Y., Léonard-Akkari L., Oulahal N., Degraeve P., Bordes C. (2019). Antibacterial properties of polyphenols: Characterization and QSAR (Quantitative Structure–Activity Relationship) models. Front. Microbiol..

[23] Ali Asgar M. (2013). Anti-diabetic potential of phenolic compounds: A review. Int. J. Food Prop..

[24] Ambriz-Prez D.L., Leyva-Lpez N., Gutierrez-Grijalva E.P., Heredia J.B. (2016). Phenolic compounds: Natural alternative in inflammation treatment. A review. Cogent Food Agric..

[25] Tounsi M.S., Ouerghemmi I., Ksouri R., Wannes W.A., Hammrouni I., Marzouik B. (2011). HPLC-Determination of phenolic composition and antioxidant capacity of cactus prickly pears seeds. Asian J. Chem..

[26] Melgar B., Dias M.I., Ciric A., Sokovic M., Garcia-Castello E.M., Rodriguez-Lopez A.D., Barros L., Ferreira I. (2017). By-product recovery of *Opuntia* spp. peels: Betalainic and phenolic profiles and bioactive properties. Ind. Crop. Prod..

[27] Dence C.W., Lin S.Y., Dence C.W. (1992). The Determination of lignin. Methods in Lignin Chemistry.

[28] Berrio-Sierra J. (2007). Etude Biochimique et Immunocytochimique de L’impact de Mutations Génétiques sur la Lignification et L’assemblage des Parois d’Arabidopsis Thaliana. Ph.D. Thesis.

[29] Shahid F., Kiritsakis A., Shahid F., Kiritsakis A. (2017). Olives and Olive Oil as Functional Foods: Bioactivity, Chemistry and Processing.

[30] Carranco N., Farrés-Cebrián M., Saurina J., Núñez O. (2018). Authentication and quantitation of fraud in extra virgin olive oils based on HPLC-UV fingerprinting and multivariate calibration. Foods.

[31] Winkelmann O., Küchler T. (2019). Reliable classification of olive oil origin based on minor component profile using 1H-NMR and multivariate analysis. Eur. J. Lipid Sci. Technol..

[32] Zenteno-Ramirez G., Juarez-Flores B.I., Aguirre-Rivera J.R., Monreal-Montes M., Merida Garcia J., Perez Serratosa M., Varo Santos M.A., Ortiz Perez M.D., Rendon-Huerta J.A. (2018). Juices of prickly pear fruits (*Opunikta* Spp.) as functional foods. Ital. J. Food Sci..

[33] Kivrak S., Kivrak I., Karababa E. (2018). Analytical evaluation of phenolic compounds and minerals of *Opuntia robusta* J.C. Wendl. and Opuntia ficus-barbarica A. Berger. Int. J. Food Prop..

[34] Kim J.W., Kim T., Yang H., Sung S. (2016). Phenolic compounds isolated from *Opuntia ficus-indica* fruits. Nat. Prod. Sci..

[35] Santos C., Campestrini L.H., Vieira D.L., Pritsch I., Yamassaki F.T., Zawadzki-Baggio S.F., Maurer J.B.B., Molento M.B. (2018). Chemical characterization of *Opuntia ficus-indica* (L.) Mill. hydroalcoholic extract and its efficiency against gastrointestinal nematodes of sheep. Vet. Sci..

[36] Allai L., Karym E.M., El Amiri B., Nasser B., Essamad A., Terzioğlu P., Ertas A., Öztürk M. (2017). Evaluation of antioxidant activity and phenolic composition of *Opuntia ficus-indica* cladodes collected from Moroccan Settat region. Eurasian J. Anal. Chem..

[37] Chahdoura H., Barreira J.C.M., Barros L., Santos-Buelga C., Ferreira I.C.F.R., Achour L. (2015). Seeds of Opuntia spp. as a novel high potential by-product: Phytochemical characterization and antioxidant activity. Ind. Crop. Prod..

[38] Talcott S.T., Passeretti S., Duncan C.E., Gorbet D.W. (2005). Polyphenolic content and sensory properties of normal and high oleic acid peanuts. Food Chem..

[39] Burri J., Graf M., Lambelet P., Löliger J. (1989). Vanillin: More than a flavouring agent—A potent antioxidant. J. Sci. Food Agri..

[40] Radnai B., Tucsek Z., Bognar Z., Antus C., Mark L., Berente Z., Gallyas F., Sumegi B., Veres B. (2009). Ferulaldehyde, a water-soluble degradation product of polyphenols, inhibits the lipopolysaccharide-induced inflammatory response in mice. J. Nutr..

[41] Zhu J., Sanidad K.Z., Sukamtoh E., Zhang G. (2017). Potential roles of chemical degradation in the biological activities of curcumin. Food Funct..

[42] Veres B. (2012). Nti-Inflammatory Role of Natural Polyphenols and Their Degradation Products.

[43] Win M.M., Abdul-Hamid A., Baharin B.S., Anwar F., Saari N. (2011). Effects of roasting on phenolics composition and antioxidant activity of peanut (*Arachis hypogaea* L.) kernel flour. Eur. Food Res. Technol..

[44] Brebu M., Vasile C. (2010). Thermal degradation of Lignin—A review. Cell. Chem. Technol..

[45] Chandrasekara N., Shahidi F. (2011). Effect of roasting on phenolic content and antioxidant activities of whole Cashew Nuts, Kernels, and Testa. J. Agric. Food Chem..

[46] Vujasinovic V., Djilas S., Dimic E., Basic Z., Radocaj O. (2012). The effect of roasting on the chemical composition and oxidative stability of pumpkin oil. Eur. J. Lipid Sci. Technol..

[47] Han F., Lu Z., Di W., Li A. (2018). Effect of roasting on phenolics content and antioxidant activity of proso Millet. Int. J. Food Eng..

[48] Cesarino I., Araújo P., Domingues-Júnior A.P., Mazzafera P. (2012). An overview of lignin metabolism and its effect on biomass recalcitrance. Braz. J. Bot..

[49] Reyes-Rivera J., Soto-Hernández M., Canché-Escamilla G., Terrazas T. (2018). Structural characterization of lignin in four cacti wood: Implications of lignification in the growth form and succulence. Front. Plant Sci..

[50] Zhao J., Xiuwen W., Hu J., Liu Q., Shen D., Xiao R. (2014). Thermal degradation of softwood lignin and hardwood lignin by TG-FTIR and Py-GC/MS. Polym. Degrad. Stab..

